# Searching for Sustainable Refrigerants by Bridging Molecular Modeling with Machine Learning

**DOI:** 10.1021/acs.iecr.2c00719

**Published:** 2022-05-18

**Authors:** Ismail I. I. Alkhatib, Carlos G. Albà, Ahmad S. Darwish, Fèlix Llovell, Lourdes F. Vega

**Affiliations:** † Research and Innovation Center on CO_2_ and Hydrogen (RICH), Khalifa University, PO Box 127788 Abu Dhabi, United Arab Emirates; ‡ Chemical Engineering Department, Khalifa University, PO Box 127788 Abu Dhabi, United Arab Emirates; § Department of Chemical Engineering, ETSEQ, Universitat Rovira i Virgili (URV), Avinguda Països Catalans 26, 43007 Tarragona, Spain

## Abstract

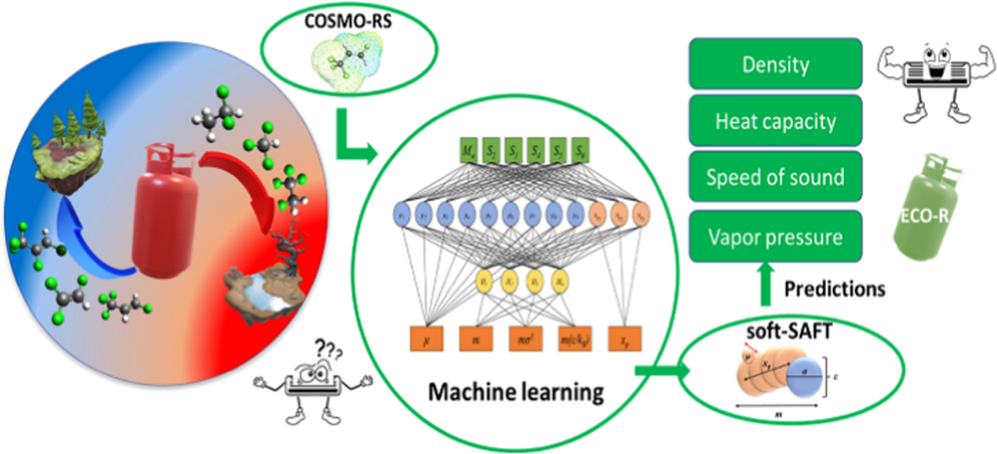

We present here a novel integrated approach employing machine learning algorithms for predicting thermophysical properties of fluids. The approach allows obtaining molecular parameters to be used in the polar soft-statistical associating fluid theory (SAFT) equation of state using molecular descriptors obtained from the conductor-like screening model for real solvents (COSMO-RS). The procedure is used for modeling 18 refrigerants including hydrofluorocarbons, hydrofluoroolefins, and hydrochlorofluoroolefins. The training dataset included six inputs obtained from COSMO-RS and five outputs from polar soft-SAFT parameters, with the accurate algorithm training ensured by its high statistical accuracy. The predicted molecular parameters were used in polar soft-SAFT for evaluating the thermophysical properties of the refrigerants such as density, vapor pressure, heat capacity, enthalpy of vaporization, and speed of sound. Predictions provided a good level of accuracy (AADs = 1.3–10.5%) compared to experimental data, and within a similar level of accuracy using parameters obtained from standard fitting procedures. Moreover, the predicted parameters provided a comparable level of predictive accuracy to parameters obtained from standard procedure when extended to modeling selected binary mixtures. The proposed approach enables bridging the gap in the data of thermodynamic properties of low global warming potential refrigerants, which hinders their technical evaluation and hence their final application.

## 1 Introduction

Our planet is witnessing one of its most notorious problems to date with the emergence of the global warming phenomenon and climate change, liable for rising atmospheric and marine temperatures, increased sea levels, extreme weather conditions, and many other environmental issues.^1^ Most of these phenomena are related to the large emissions of greenhouse gases (GHGs) into the atmosphere. Chlorofluorocarbons (CFCs), known as 2nd generation refrigerants, widely used in refrigeration and cooling applications since the 1930s, were banned in the Montreal’s Protocol,^2^ due to their highly destructive effect on the ozone layer,^3−5^ and were replaced by hydrofluorocarbons (HFCs), with zero ozone depletion potential (ODP). However, HFCs are known as potent GHGs, with high global warming potential (GWP), and are to be phased out due to the Kigali Amendment to Montreal’s Protocol.^6^ The gradual phase-out of HFCs under current environmental legislations led to the search for alternative sustainable refrigerants anew, focusing on desirable properties including the benign environmental effect (*i.e.*, null ODP, low GWP, and short atmospheric lifetime), an acceptable level of safety (*i.e.*, low toxicity and flammability), and excellent technical performance.^2,7^ For a while, the search for 4th generation refrigerants remained elusive,^8^ until the principles of green chemistry and engineering came to the rescue,^9^ identifying hydrofluoroolefins (HFOs), hydrochlorofluoroolefins (HCFOs), hydrofluoroethers (HFEs), and blends of natural or synthetic refrigerants as potential alternatives.^10−12^

Hence, refrigerants to replace the high-GWP ones currently used must meet three criteria: (1) environmentally friendly, (2) safety requirements, and (3) excellent technical performance. The first two criteria are inherent to the pure refrigerant depending on their atomic constituents and structure, acting as the first layer of screening by discarding those unable to meet environmental and safety standards. In contrast, the third criterion relies on detailed knowledge of a refrigerant’s thermodynamic properties required for accurate process design and evaluation. This is the main hurdle in the commercialization of newly developed refrigerants achieving the environmental and safety requirements, given the large number of thermodynamic properties required for accurate technical evaluation.^13^

The monumental work of McLinden et al.^11^ exemplifies the difficulties associated with the search for greener alternative refrigerants. Their systematic screening of a large array of possible substances from available databases and experimental datasets resulted in a pool of 138 pure candidate refrigerants meeting the required environmental and safety limits. The additional technical performance assisted in the identification of 27 possible low-GWP single-component refrigerants finely balancing the trade-offs between the aforementioned two selection criteria. Such an effort was facilitated by the availability of extensive databases on the relevant properties of known substances studied over the past 20 years. However, this becomes increasingly difficult in the case of newly developed green refrigerants, as the standard experimental route to obtaining property measurements has long ceased to be capable of meeting the exponentially growing number of newly developed refrigerants and blends. This has accelerated the increasing need for predictive computational modeling tools for evaluating the thermodynamic behavior of these refrigerants and obtaining their relevant properties required for technical evaluation.^14−16^ Of particular prominence are molecular modeling techniques, bridging microscopic characteristics of a fluid with its observable macroscopic properties, becoming indispensable for accurately predicting thermodynamic properties and enhancing the fundamental understanding of complex systems.^15,17−20^ Outstanding contributions have been made in this field. For instance, Raabe and co-workers^21−26^ employed classical molecular simulations to predict the thermophysical properties of pure HFOs and HCFOs, for which few experimental data were available. Recently, García et al.^27^ performed a computational screening of 40 experimentally available metal–organic frameworks (MOFs) exploring their application in adsorption air-conditioning with selected low-GWP refrigerants, searching for the optimal MOF–refrigerant pair. The computational study provides useful guidance on the MOF topology, composition, and pore sizes needed to achieve the best performance with the selected low-GWP refrigerants for this application. Mambo–Lomba and Paricaud^28^ developed a thermodynamic model for different pure refrigerants and their binary mixtures, based on different versions of the conductor-like screening model for real solvents (COSMO-RS).^29,30^ Vega and co-workers^31−38^ employed different versions of the statistical associating fluid theory (SAFT)^39^ equation of state (EoS), namely, the Lennard–Jones version (soft-SAFT) and polar perturbed chain version (PC-SAFT), to study a wide range of thermodynamic, interfacial, and transport properties of pure HFCs, HFOs, and HCFOs demonstrating the efficacy of molecular modeling in the search for alternative eco-friendly materials.^40^ Polishuk et al.^41^ implemented PC-SAFT and the variable attractive range SAFT version (SAFT-VR) to study the thermodynamic properties of 3rd generation HFCs and their mixtures. Vinš et al.^42^ developed a property model using PC-SAFT for the density and surface tension of 4th generation HFEs. Mickoleit et al.^43^ examined the effect of the thermodynamic model on the accuracy of screening and designing refrigeration systems, examining models including classical cubic equations of state, COSMO-based models, and PCP-SAFT. Yang et al.^44^ applied entropy scaling with the REFPROP^45^ reference EoS to model the thermal conductivity of several pure and mixed refrigerants. Wang et al.^46^ integrated a polar version of PC-SAFT with process modeling and optimization to determine optimal refrigerants based on technical evaluation.

Despite this, it remains difficult to assert the adequacy of one thermodynamic model over another for property predictions for emerging green refrigerants, as all of these models suffer from some limitations, with no singular universal model. For example, molecular simulations are computationally expensive for force-field parametrization and data generation over a wide range of operating conditions of interest, as required for the detailed characterization of these compounds. Conversely, the accuracy of purely predictive COSMO-based models is qualitative rather than quantitative, which can be enhanced by adjusting some of the model’s parameters at the expense of losing its fully predictive nature.^28^ Finally, SAFT-based EoSs require the availability of experimental data,^47^ even if somewhat limited in terms of type and range, to regress the parameters descriptive of the pure refrigerant. Granted these parameters can be transferable across molecular families with specific analoguous molecular characteristics,^48^ yet this approach is not fully foolproof when applied to new and emerging refrigerants with unavailable data.

Conversely, the combination of different modeling approaches exploiting the synergetic benefits of several modeling tools has proved to be a beneficial endeavor for accelerating the development of molecular modeling tools for assessing emerging low-GWP refrigerants, even in the absence of data. Raabe^49^ coupled molecular simulations and PC-SAFT to study the phase equilibria of some 4th generation refrigerants with 3rd generation refrigerants and CO_2_. Similarly, Fouad and Alasiri^50^ employed molecular dynamics simulation to generate data for the viscosity and self-diffusion coefficient required for regressing parameters of polar PC-SAFT of selected low-GWP refrigerants. Moreover, Li et al.^51^ employed molecular dynamics simulation and polar PC-SAFT to examine the interfacial anomaly for selected refrigerant blends of HFOs and *n*-alkanes. These contributions relied on molecular simulations for data generation in the absence of experimental data. The simulation data was then used to parametrize the more rapid molecular-based EoSs, enabling extrapolation of property measurements at industrially relevant conditions without incurring high computational costs.

Furthermore, the recent rise of the 4th industrial revolution paved the path for integrating artificial intelligence (AI) and machine learning with molecular modeling approaches for a number of applications including the rational design of new green materials, predicting thermodynamic behavior of complex systems, and accelerating the development of force-fields for molecular simulations.^52^ The integration of machine learning with other molecular modeling approaches such as COSMO-RS has found success in predicting the thermodynamic behavior of several classes of green materials as shown in refs (53−62). Therefore, it can be expected that such integration with molecular-based EoSs will exploit their rapid, accurate, and holistic modeling features for obtaining the thermodynamic properties of emerging green refrigerants, required for their technical assessment.

In this contribution, we present for the first time a novel paradigm for predicting molecular parameters of pure refrigerants, modeled using polar soft-SAFT EoS,^63^ applied to eighteen 3rd and 4th generation refrigerants including HFCs, HFOs, and HCFOs, provided in Table 1. These refrigerants were chosen based on two features. First, the availability of experimental data required to characterize these fluids using polar soft-SAFT and providing the training dataset. Second, the 3rd generation refrigerants were those currently used in the market, while the 4th generation refrigerants were those with demonstrated potentiality as sustainable alternatives with excellent environmental performance. The developed framework is built on using machine learning algorithms, specifically, artificial neural network (ANN), to predict polar soft-SAFT parameters using molecular descriptors obtained from COSMO-RS. In this manner, even in the absence of experimental data for polar soft-SAFT EoS parametrization, the coarse-grain representation of a pure fluid can be easily obtained from the predictive power of COSMO-RS and machine learning. With these parameters, the holistic modeling capability of polar soft-SAFT can be fully exploited to characterize the desired fluids. This work is a demonstration of the usefulness of the approach for a specific relevant need, and a step forward toward a more predictive framework for estimating SAFT-based parameters in the search for fluids for different industrial applications.

**Table 1:** List of 3rd and 4th Generation Refrigerants Included in This Work

fluoromethane (R41)	1,1,1,2,2-pentafluoroethane (R125)	2,3,3,3-tetrafluoroprop-1-ene (R1234yf)
difluoromethane (R32)	1,1,1,3,3-pentafluoropropane (R245fa)	*trans*-1,3,3,3-tetrafluoroprop-1-ene (R1234ze(E))
trifluoromethane (R23)	1,1,1,3,33-hexafluoropropane (R236fa)	*cis*-1,2,3,3,3-pentafluoroprop-1-ene (R1225ye(Z))
1-fluoroethane (R161)	1,1,1,2,3,3-heptafluoropropane (R227ea)	*cis*-1,1,1,4,4,4-hexafluoro-2-butene (R1336mzz(Z))
1,1-difluoroethane (R152a)	1,1,2-trifluoroethene (R1123)	*trans*-1-chloro-3,3,3-trifluoroprop-1-ene (R1233zd(E))
1,1,1,2-tetrafluoroethane (R134a)	3,3,3-trifluoroprop-1-ene (R1243zf)	*cis*-1-chloro-2,3,3,3-tetrafluoroprop-1-ene (R1224yd(Z))

## 2 Methodology

**Figure 1:**
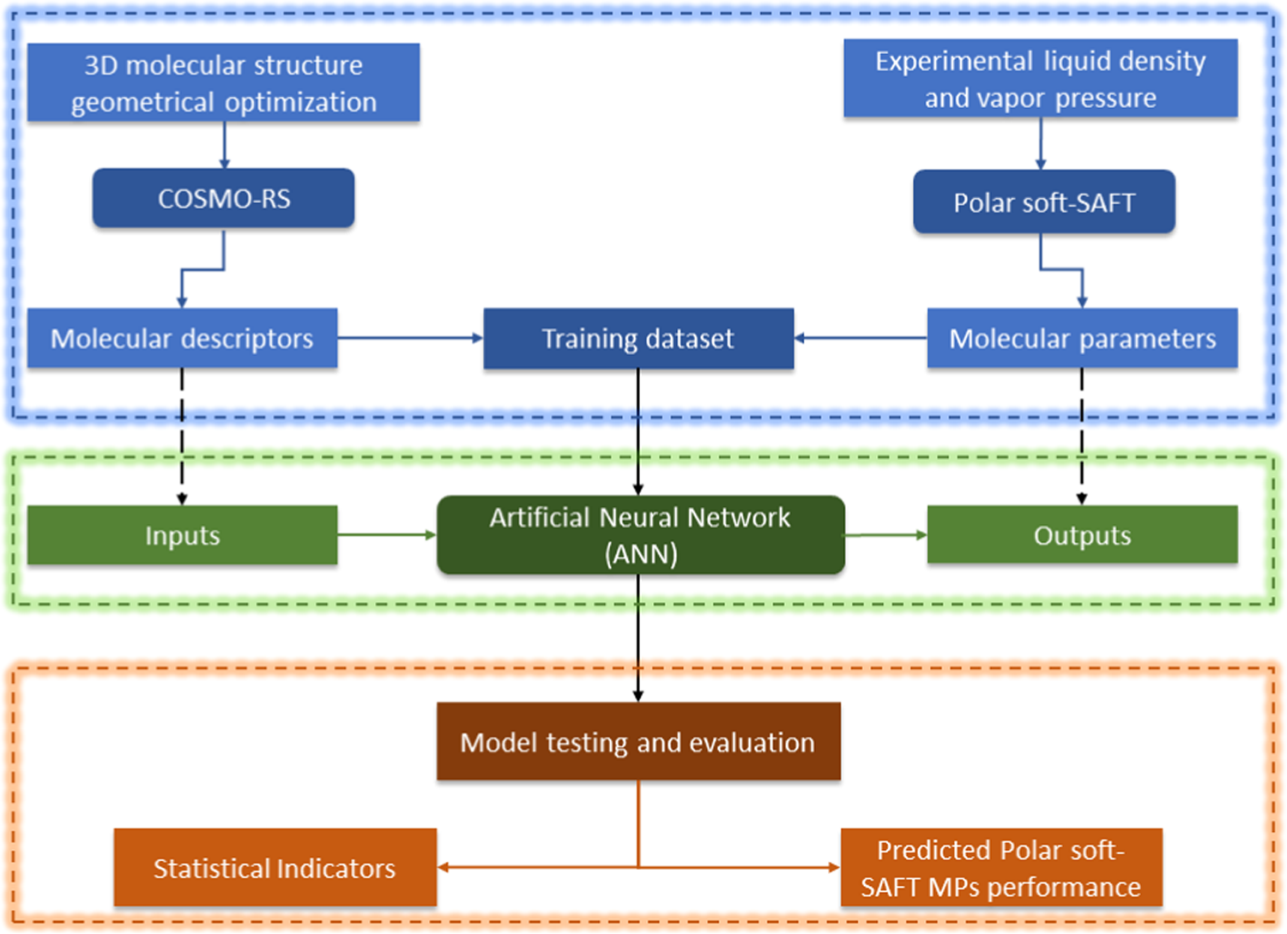
Schematic of the integrated modeling framework developed in this work.

The general paradigm of the methodology adopted in this work, highlighted in Figure 1, involves three stages. The first stage relies on using molecular modeling tools, COSMO-RS, and polar soft-SAFT, to generate the dataset required for ANN training. Subsequently, the ANN model is developed to establish a link between COSMO-RS molecular descriptors (inputs) and polar soft-SAFT molecular parameters (outputs) for the eighteen studied refrigerants. Finally, the trained ANN model is evaluated based on statistical indicators and used to assess the adequacy of using ANN-predicted polar soft-SAFT parameters in modeling the thermodynamic behavior of the studied refrigerants.

### 2.1 COSMO-RS Molecular Descriptors

**Figure 2:**
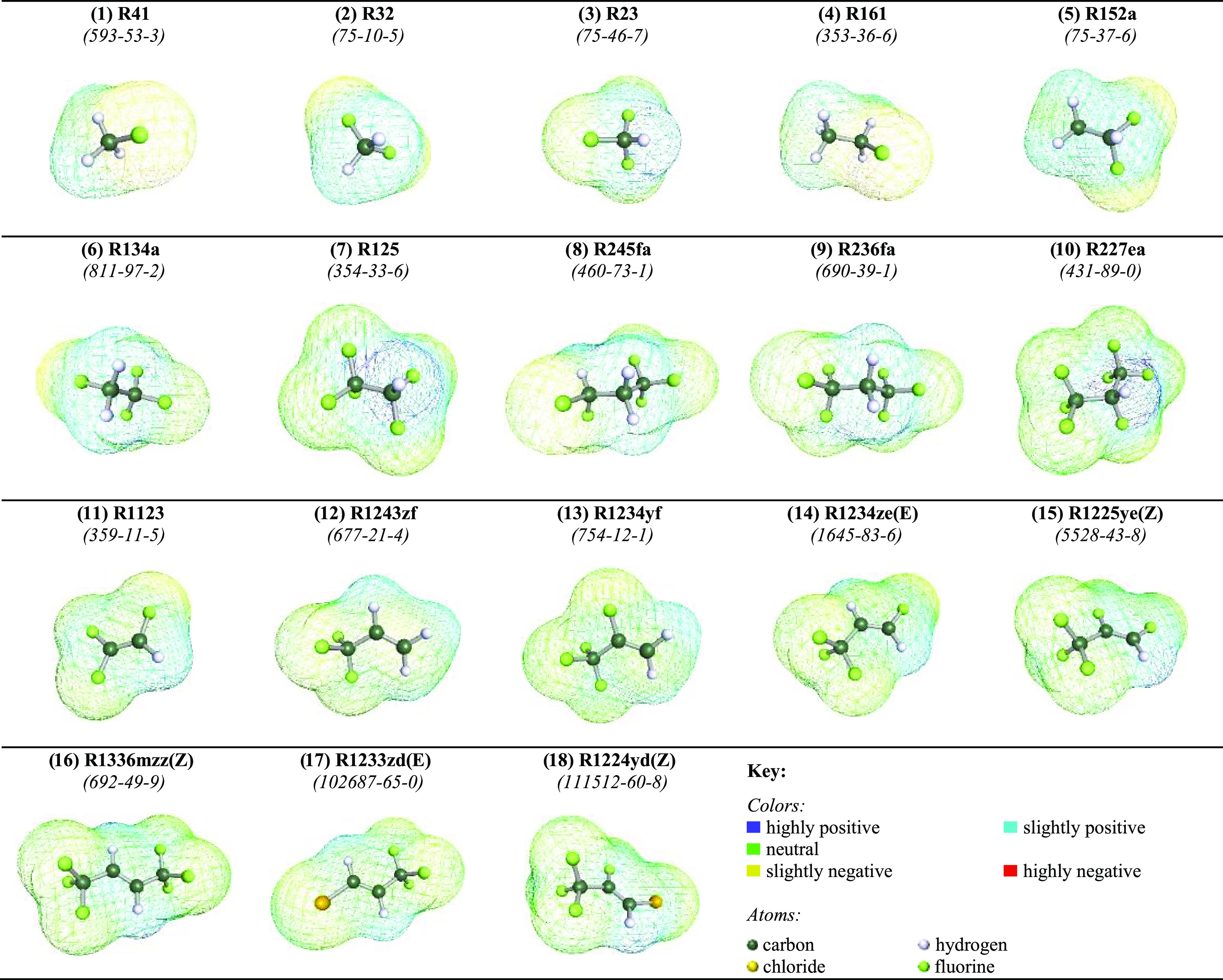
Geometrically optimized 3D COSMO-RS surfaces of the 18 modeled refrigerants. The CAS# for each refrigerant is highlighted in italics.

In this work, COSMO-RS is used to obtain molecular descriptors representative of the studied refrigerants, used as inputs into the ANN model. With COSMO-RS,^29,30^ the surface charge density of a molecule, obtained from density functional theory (DFT), is transformed into discrete surface segments with a specific screening charge density (σ), incorporating contributions from different intermolecular interactions. This generates the so-called σ-profile and σ-potential, representing the probability of specific charge density on a surface segment, and the affinity to a specific polarity surface, respectively.^29,30^ To do so, the three-dimensional (3D) molecular structures for the eighteen refrigerants were built using Turbomole software (TmoleX version 4.5.1)^64^ and then geometrically optimized at the DFT level using BP86 functional with the def-TZVP basis set, as provided in Figure 2. The optimized structures were used to obtain the σ-profiles for each refrigerant using COSMO-RS software (COSMOThermX version 19.0.5).^65^ The generated σ-profiles were then discretized into eight regions, each with a screening charge density of 0.00625 e/Å^2^, used to compute the molecular descriptors namely, *S*_σ-profile_, as integrals of the area under the σ-profile curves in those eight regions.^66^ Though the σ-profiles can be divided into 51 regions for more accurate models,^30^ it was opted to maintain the relative simplicity of the model by minimizing the number of descriptors. The advantage of using these molecular descriptors as ANN inputs is that aside from being obtained *a priori* without fitting, they also contain sufficient information indicative of the structural and energetic nature of the molecule, needed to predict its governing intermolecular interactions. Additional details on the implementation of COSMO-RS to obtain σ-profiles and their discretization can be found elsewhere.^55,66^

### 2.2 Polar Soft-SAFT Molecular Parameters

Polar soft-SAFT is used to obtain molecular parameters descriptive of the studied refrigerants for their holistic thermodynamic evaluation, used as outputs into the ANN model. With polar soft-SAFT,^63^ the residual Helmholtz energy density (*a*^res^) of a pure fluid is computed as the sum of various microscopic contributions
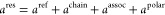
1Each term in the equation explicitly accounts for different structural and energetic features of the fluid. The reference term (*a*^ref^) represents the repulsive and dispersive interactions between the individual Lennard–Jones (LJ) group integrating the molecule (also called segments in the SAFT nomenclature). The chain term (*a*^chain^) represents the chain formation from connected LJ segments. The association term (*a*^assoc^) denotes highly directional and short-range intermolecular interactions such as hydrogen bonding. Finally, the polar term (*a*^polar^) stands for the explicit contribution from multipolar intermolecular interactions such as a permanent dipole or quadrupole. Additional details on the theory and mathematical expressions for each term can be found in the original publications.^63,67^

**Figure 3:**
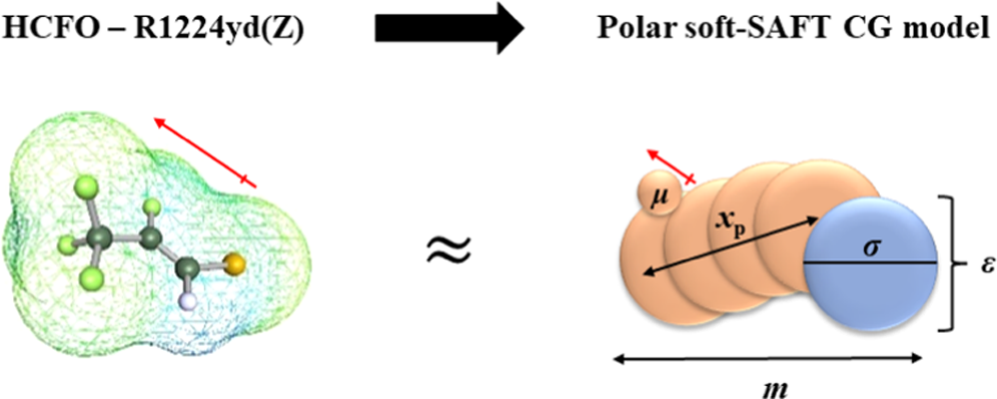
Molecular structure of HCFO R1224yd(Z) as a representative of one of the refrigerants studied in this work and its equivalent polar soft-SAFT coarse-grain molecular model.

Applying the theory to pure fluids requires proposing a simplified coarse-grain model representative of the key molecular features (*i.e.*, structure, energy, interactions, *etc.*) captured through a set of molecular parameters. The eighteen refrigerants included in this work were modeled as non-associating LJ chainlike fluids with explicit consideration of their permanent dipole moments, arising from the presence of halogen atoms in their structure, leading to asymmetrical charge distribution. As such, five molecular parameters were required to characterize each refrigerant, including LJ segment diameter (σ), chain length (*m*), LJ segment’s dispersive energy (ε), dipole moment (μ), and the fraction of dipolar segments (*x*_p_), as shown in Figure 3. To ensure a robust model and minimized fitting to data, the dipole moment was fixed to the experimental value in a vacuum, and the fraction of dipolar segments was determined *a priori* based on a physical argument highlighted in previous contributions.^63,68−70^ With that, only the three remaining parameters (*i.e.*, *m*, σ, ε) were simultaneously fitted to limited data sets including each refrigerant’s liquid density and vapor pressure.

Notice that once the molecular parameters of a molecule are available, polar soft-SAFT can be used to calculate thermodynamic properties of that particular fluid for a wide range of conditions. In addition to vapor–liquid equilibria, these parameters can be used to predict other thermodynamic information needed for characterizing the refrigerants, such as heat capacity, speed of sound, single-phase density, enthalpy of vaporization, *etc.*, and extended to model multicomponent mixtures.^35,36,38^ All thermodynamic calculations performed using polar soft-SAFT have been performed using the proprietary code of Vaga and co-workers developed and updated over the past 20 years.^71^

### 2.3 ANN Algorithm

To predict polar soft-SAFT molecular parameters descriptive of the thermodynamic behavior of pure refrigerants, a feed-forward ANN is used to map the relation between COSMO-RS molecular descriptors and polar soft-SAFT molecular parameters, in the absence of experimental data for parametrization. The network is composed of three layers: (1) the input layer with the refrigerant molecular weight, and eight COSMO-RS molecular descriptors, (2) hidden layer, with several processing neurons, and (3) output layer with the five polar soft-SAFT molecular parameters. The neurons in each layer are interconnected *via* direct links with their associated weights, representing the information used to map the relation between inputs and outputs. The hyperbolic tangent sigmoid activation function is used to connect input neurons (*n*_*i*_) to hidden neurons (*H*_*j*_), while the output neurons (*O*_*k*_) are linearly connected to the hidden neurons, as^72^
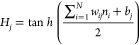
2a
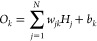
2bwhere, *w*_*ij*_ and *w*_*jk*_ are the weights for links of type [input neuron *i*—hidden neuron *j*] and [hidden neuron *j*—output neuron *k*], respectively, while *b*_*j*_ and *b*_*k*_ are the biases of hidden neuron *j* and output neuron *k*, respectively.

The ANN model was developed using the neural networks toolbox of the John’s Macintosh Project (JMP) statistical software,^73^ with the supervised training task done using the training dataset pairs of COSMO-RS descriptors and polar soft-SAFT parameters for fifteen refrigerants while the three remaining refrigerants, namely, R134a, R32, and R1243zf, were used for internal validation. It is granted that the size of the dataset is relatively small compared to other applications employing machine learning, however, this is sufficient for a proof of concept, as will be shown later.

Several statistical indicators were used to evaluate the developed ANN model including coefficient of determination (*R*^2^), absolute average relative deviation (AARD), root mean square error (RMSE), and average standard deviation (SD_avg_), for each output. Nevertheless, the most crucial evaluation of the developed ANN model is by utilizing its predicted polar soft-SAFT molecular parameters in characterizing the thermodynamic behavior of the three refrigerants not included in the training set and checking their accuracy compared to experimental data.

## 3 Results and Discussion

### 3.1 σ-Profile and Molecular Descriptors of Selected Refrigerants from COSMO-RS

**Figure 4:**
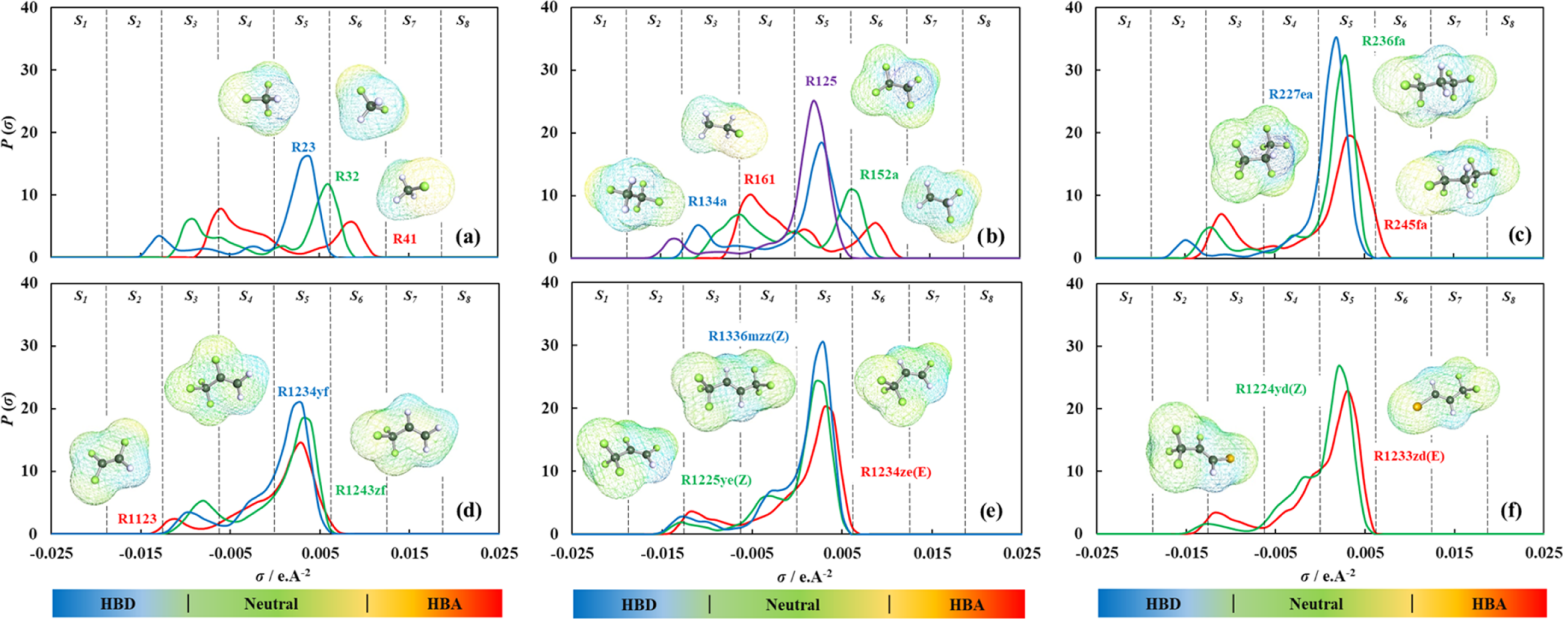
COSMO-RS calculated σ-profiles for the 18 refrigerants with (a) HFCs with 1 carbon, (b) HFCs with 2 carbons, (c) HFCs with 3 carbons, (d) and (e) HFOs, and (f) HCFOs. See Figure 2 for key structural details of each refrigerant.

Based on the aforementioned approach in Section 2.1, and the geometrically optimized 3D structure for each pure refrigerant in Figure 2, the discretized σ-profiles are provided in Figure 4. From the σ-profiles it is inferred that the polarity surfaces are generally within three distinct regions, with negative charge densities (σ < −0.01 e/Å^2^) denoting positive polarity surfaces with a hydrogen donating character, while positive charge densities (σ > +0.01 e/Å^2^) denoting negative polarity surfaces with a hydrogen accepting character. Meanwhile, charge densities within the range of −0.01 ≤ σ ≤ +0.01 e/Å^2^ represent neutral nonpolar surfaces within the molecule. The discretization of the σ-profiles into the eight molecular descriptors highlighted earlier with a σ step size of 0.00625 e/Å^2^, provides insight into the atomistic nature of each molecule and their contribution to its governing intermolecular interactions based on the location, height, and width of the peaks. For example, for HFCs with one carbon atom, such as R41, R32, and R23, seen in Figure 4a, the left-side skewness of their σ-profile increases with increased fluorine atoms in the molecule, making their hydrogens more positively polarized (transition from *S*_4_ to *S*_2_ with decreasing peaks), while their fluorine-less counterparts negatively polarized (transition from *S*_6_ to *S*_5_ with increasing peaks). The same is observed for HFCs with 2 or 3 carbon backbones, as shown in Figure 4b,c, respectively. For HFOs in Figure 4d,e, and HCFOs in Figure 4f, the small less distinct peaks seen in the slightly positive polarity region (*S*_4_) can be attributed to the double-bonded carbons in their structure. For all examined refrigerants, no distinct peaks were observed in strongly positive polarity (*S*_1_), and strongly negative polarity (*S*_7_ and *S*_8_), as these regions are attributed to ions (H^+^, F^–^, Cl^–^, *etc*.), as such, these descriptors were removed from the training dataset. In some cases, such as those for R23, R125, and R227ea, the small probability of strongly positive polarizable atoms shown from the small peaks in (*S*_2_) was attributed to the effect of the halogens on polarizing the single hydrogen in their molecular structure. The ANN inputs dataset including molecular weight and the five COSMO-RS descriptors (*S*_2_, *S*_3_, *S*_4_, *S*_5_, *S*_6_) are provided in Table S1 in the Supporting Information (SI).

### 3.2 Polar Soft-SAFT Molecular Parameters: Physical Meaning and Their Role in the Dataset

The second part of the dataset development for ANN training is obtaining the polar soft-SAFT parameters for the eighteen selected refrigerants. The molecular parameters descriptive of the modeled refrigerants using polar soft-SAFT were transferred from earlier contributions based on the aforementioned parametrization approach,^36,38^ with the parameters included in Table S2 in the SI. The robustness of these molecular parameters was rigorously tested by predicting other thermodynamic properties not included in the fitting process, comprising single-phase density, isobaric heat capacity, enthalpy of vaporization, and speed of sound, along with predictive extension of the model to binary mixtures with *n*-alkanes.^38^

Here, we will focus on evaluating their physical meaning as it can help in assessing their validity and possible transferability for similar compounds, and hence, their role in the dataset. The nonpolar parameters (*i.e.*, *m*, σ, ε) can be correlated to the molecular size, volume, and energy of each refrigerant, with *m*σ^3^ representing the volume of the molecule, while *m*(ε/*k*_B_) stands for its dispersive energy. As such it was opted to construct the polar soft-SAFT output dataset with five parameters (μ, *x*_p_, *m*, *m*σ^3^, *m*ε/*k*_B_) to ensure meaningful physically oriented trends, included in Table S3 in the SI.

**Figure 5:**
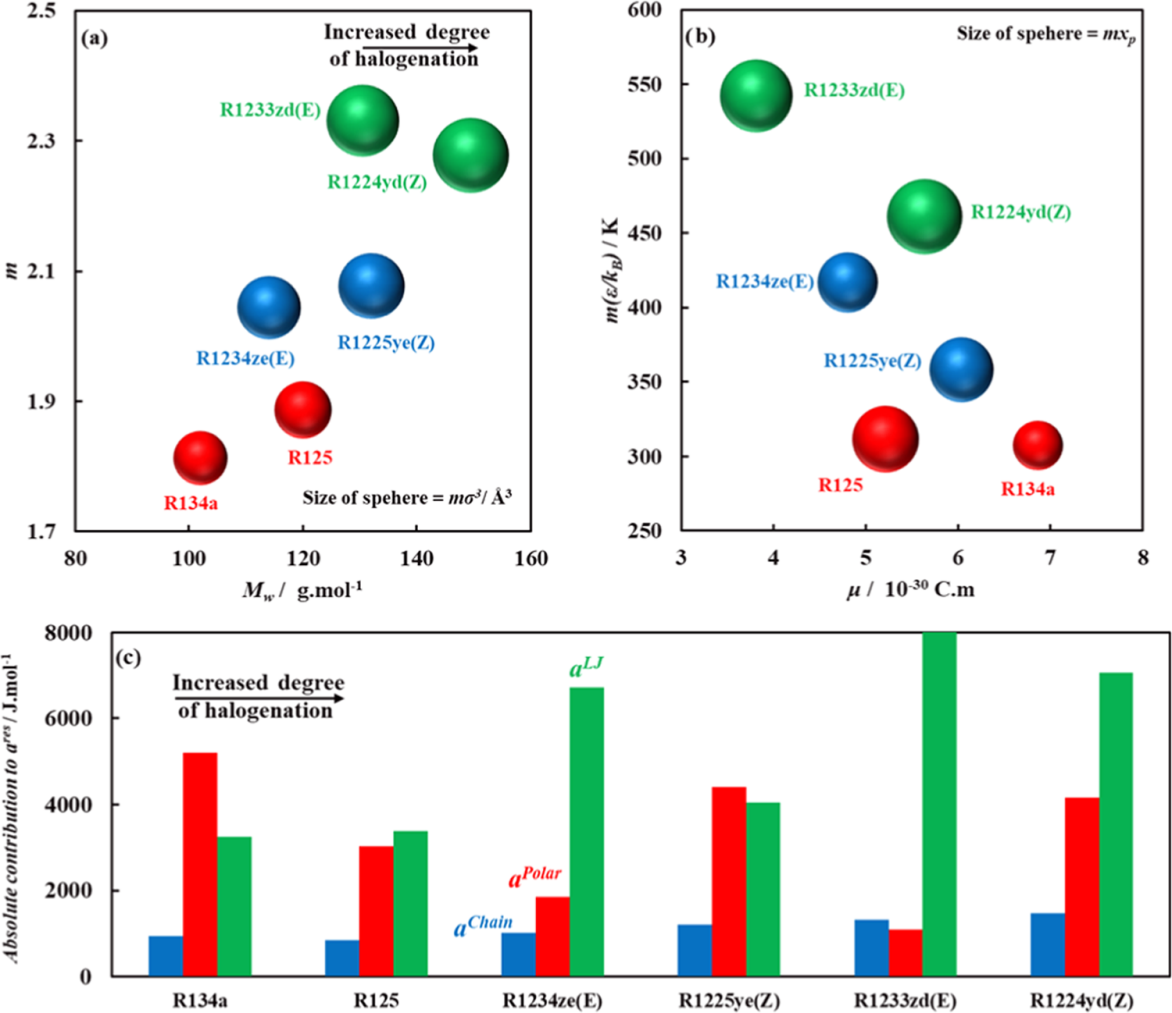
Comparative analysis of polar soft-SAFT CG model parameters for the selected refrigerants in terms of the (a) effect of molecular weight on the chain length and molecular volume of the refrigerants (represented by the size of the sphere), (b) dipole moment versus LJ molecular energy of the refrigerants and polar segments, *mx*_p_ (represented by the size of the sphere), and (c) polar soft-SAFT predicted absolute contributions of different terms to residual Helmholtz energy at *T* = 250 K (eq 1 with *a*^assoc^ = 0). The values of the parameters are provided in Table S3 in the SI.

For the sake of highlighting the meaning of these parameters, we present the analysis of selected HFCs (two-carbon chain: R134a, and R125), HFOs (three-carbon chain: R1234ze(E), R1225ye(Z)), and HCFOs (three-carbon chain: R1233zd(E), R1224yd(Z)) to isolate the effect of carbon number and degree of halogenation on the structure and energy of the refrigerants, as provided in Figure 5 (see Figure 2 for the 3D molecular structure). In terms of the molecular size of each refrigerant, the increased degree of halogenation for the selected HFCs and HFOs was associated with increased molecular chain length and molecular volume (*m*σ^3^) as shown in Figure 5a. This partially holds true for HCFOs, where the increased halogenation resulted in increased molecular volume, but not in an increase in the chain length. Notice also that the effect of increasing carbon chain length from the two-carbon backbone HFCs to three-carbon backbone HFOs/HCFOs resulted in similar trends, consistent with the increased molar mass of these refrigerants. Similarly, the replacement of chlorine in HCFOs with the fluorine in their counterpart HFOs (*i.e*., R1233zd(E) *vs* R1234ze(E), and R1224yd(Z) *vs* R1225ye(Z)) has a larger impact on increasing the molecular volume of the refrigerant associated with chlorine’s higher atomic weight. These trends are also supported by the increased contribution of the chain term (*a*^chain^) to the residual Helmholtz energy of the molecules (*a*^res^), obtained from eq 1 at *T* = 250 K, shown in Figure 5c.

Regarding the molecular parameters representing energetic contributions (*i.e.*, *m*(ε*/k*_B_), μ, *x*_p_), two opposing trends are observed, depending on the degree of saturation of the carbon backbone, as highlighted in Figure 5b. For fully saturated HFCs, increasing the degree of fluorination was associated with a reduction in the magnitude of the dipole moment, albeit increased effective polar segments (*mx*_p_). This is responsible for R125 lower polar contribution compared to R134a, as seen in Figure 5c, with increasing number of fluorine atoms in the molecular structure contributing toward increasing symmetry of the charge distribution. On the opposite side, increasing the degree of halogenation for HFOs and HCFOs, resulted in a reduction in the dispersive energy, accompanied by the increased magnitude of the dipole moment and polar segments. This is also seen from the increased polar contribution and reduced reference term contribution (LJ interactions) to the residual Helmholtz energy shown in Figure 5c. This can be attributed to the presence of double-bonded carbons in these molecules, being polarizable, as opposed to saturated carbon bonds, contributing to the charge asymmetry for these refrigerants, as also seen from the small peaks at *S*_4_ in their σ-profiles in Figure 4. Additionally, notice the effect of replacing fluorine with chlorine (HFOs *vs* HCFOs), reducing the polarity of the HCFOs and their polar contributions, due to the lower electronegativity of chlorine atoms.

### 3.3 ANN Model Performance and Evaluation

**Figure 6:**
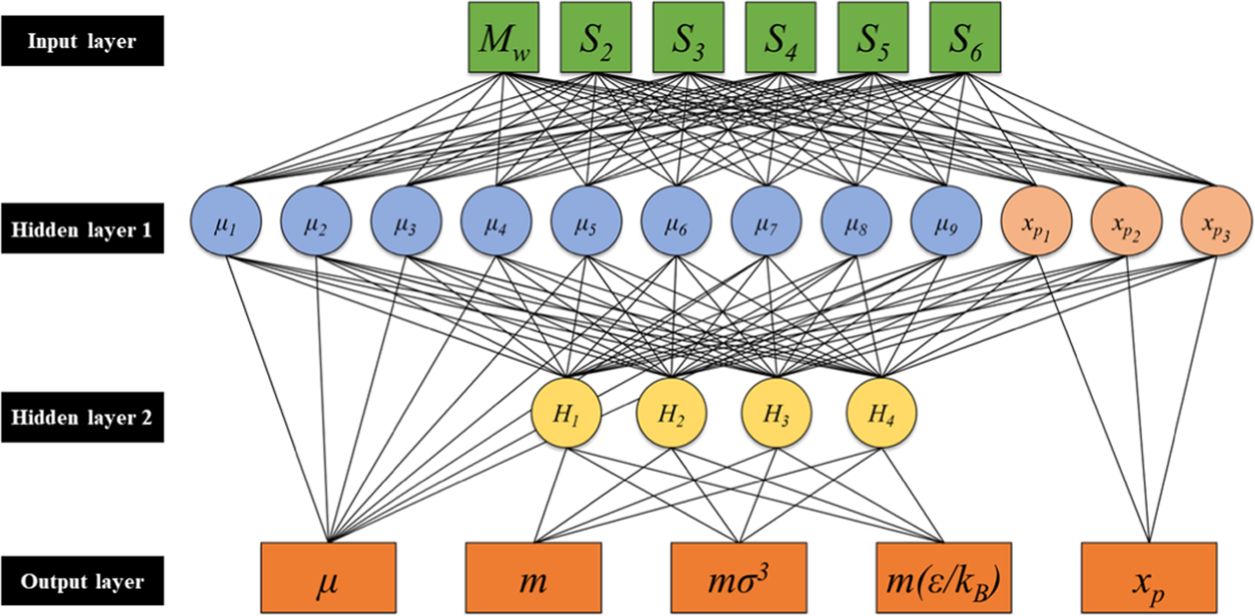
Integrated 6–9–1, 6–3–1, and 6–12–4–3 ANN to predict polar soft-SAFT molecular parameters for the studied refrigerants.

With the datasets for the eighteen refrigerants obtained using the aforementioned molecular modeling approaches (COSMO-RS and polar soft-SAFT), the ANN model for predicting polar soft-SAFT molecular parameters was developed, with the network topography shown in Figure 6. The construction of the best-performing network, out of the several options developed in earlier stages (not shown here), required two hidden layers. The first hidden layer with 12 neurons for computing the fraction of polar segments (*x*_p_), and dipole moment (μ) of the molecule, with 3 and 9 neurons for each, respectively. The second hidden layer receives inputs from the neurons in the first hidden layer, into 4 neurons for computing the remaining polar soft-SAFT parameters such as chain length, volume, and LJ energy of the molecule. The network was trained with data for 15 refrigerants, while the remaining three (*i.e.*, R32, R134a, and R1243zf) were used for testing the trained ANN model. Overall, the developed network required 120 weights, and 16 biases, to correlate COSMO-RS molecular descriptors with polar soft-SAFT molecular parameters of the studied refrigerants. The associated weights and biases for all links between neurons in each layer are provided in Tables S4–S6 in the SI for completeness.

The architecture of the developed network was chosen to segregate the molecular parameters applicable for all molecular families (*i.e.*, dispersive, associating, polar, *etc*.) from those specific for polar fluids (*i.e.*, μ, *x*_p_), within the same integrated network. This framework has the possibility of flexible future application and extension to other molecular families with different chemical nature than the dipolar refrigerants included in this work. Additionally, during the ANN model development, it was established that the number of hidden neurons in each processing layer was decisive in controlling the accuracy and complexity of the model. The number of neurons in each layer were incrementally changed up to the number of critical neurons in each layer without substantial improvement in modeling accuracy while maintaining a relatively simple ANN architect.

**Figure 7:**
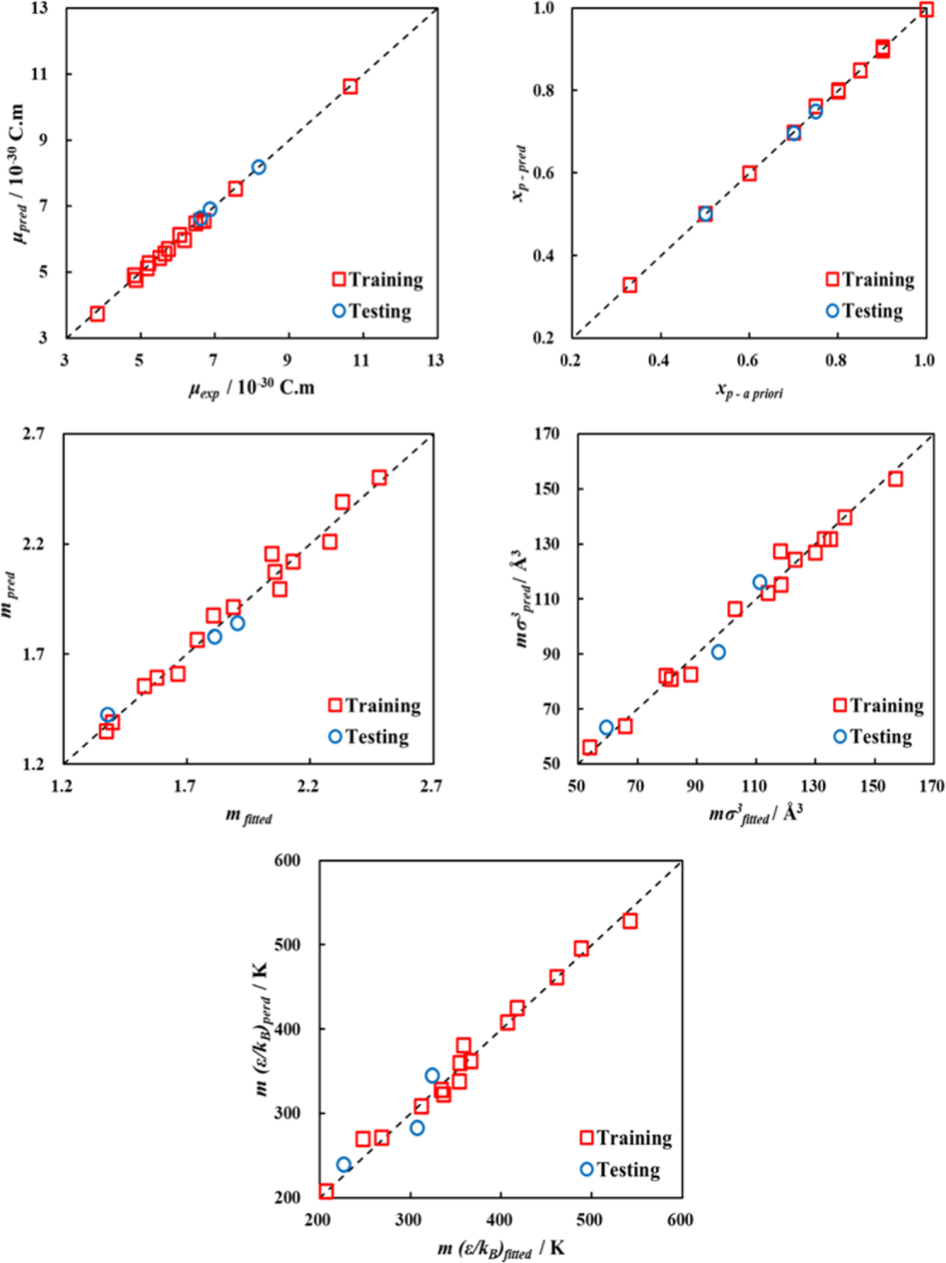
Parity plots of fitted *vs* ANN-predicted polar soft-SAFT parameters for training and testing dataset.

Provided in Table 2 is a summary of the performance of the training and testing of the developed ANN model based on statistical indicators. The accurate training of the ANN model can be established from its high *R*^2^ > 0.99, and narrow dispersion (*i.e.*, small RMSE), especially for μ, and *x*_p_ parameters due to the high number of processing neurons, used in their correlation. Conversely, for the remaining parameters, the goodness of fit is with a good level of accuracy (*R*^2^ > 0.97) and an acceptable level of dispersion for some data points. This can also be visually supported from the parity plots shown in Figure 7 comparing ANN-predicted polar soft-SAFT parameters (*y*-axis) and those obtained using the standard parametrization approach, fitting to experimental data (*x*-axis). It should be noted that the higher values of the statistical indicators for training and testing ANN for *m*(ε/*k*_B_) output parameter are associated with its larger magnitude. However, these results indicate the proper training of the developed model and its ability to map the relation between the chosen inputs and outputs. The subsequent testing of the model’s external predictive power using refrigerants R32, R134a, and R1243zf, also shown in Table 2 and Figure 7, attests to its high predictive accuracy supported by the statistical indicators and acceptable agreement between predicted and fitted parameters for those refrigerants.

**Table 2:** Statistical Performance of ANN Model Training and Testing for Each Network Output

indicator	*R*^2^		AARD%		RMSE		SD_avg_	
parameter	training	testing	training	testing	training	testing	training	testing
μ·[10^–30^ C·m]	0.9970	1.0000	1.32	0.49	0.0899	0.0355	0.05	0.02
*x*_p_	0.9996	0.9998	0.31	0.24	0.0038	0.002	0.00	0.00
*m*	0.9786	0.9994	2.15	2.92	0.0510	0.050	0.03	0.03
*m*σ^3^ [Å^3^]	0.9847	0.9452	2.72	5.97	3.561	5.229	1.97	3.64
*m*(ε/*k*_B_) [K]	0.9841	0.7981	2.51	6.78	11.150	20.170	6.05	13.87

To better elucidate the impact of ANN accuracy on predicting molecular parameters for molecular theory, the ANN-predicted parameters were utilized to predict the vapor pressure and saturated liquid density of the pure refrigerants using polar soft-SAFT compared to experimental property data^45,74−82^ quantified in terms of absolute average deviation (AAD). As highlighted in Table 3, the standard parametrization has a higher accuracy in predicting the thermodynamic properties of pure refrigerants, with an average AAD of 1.899, and 0.405% for vapor pressure and saturated liquid density, respectively. Although the training of the ANN model showed high accuracy (see Table 2), the application of the ANN-predicted parameters to molecular theory was associated with higher deviations from experimental data than those seen earlier for parameters from standard procedure, with average AADs of 4.753 and 1.539% for vapor pressure and saturated liquid density, respectively. The higher deviation obtained for vapor pressure is closely linked to the lower correlatability of COSMO-RS molecular descriptors with the dispersive energy of the molecules (*m*ε/*k*_B_), in Table 2, although the σ-profile molecular descriptors are more descriptive of structural features rather than energetic ones, they provide sufficient details to obtain an acceptable mapping relationship between the inputs and outputs.

**Table 3:** AAD for Vapor Pressure and Saturated Liquid Density between Polar Soft-SAFT Predictions and Experimental Data for the Pure Refrigerants^a^

	standard parametrization^36,38^		ANN predicted		
compound	AADP* [%]^b^	AADρ [%]^c^	AADP* [%]^b^	AADρ [%]^c^	*T* range [K]
Training Set					
R41	1.494	0.763	7.110	1.068	200–280
R23	1.327	0.115	1.388	1.629	200–270
R161	1.374	0.414	2.678	2.403	200–340
R152a	0.993	0.792	5.259	2.607	200–356
R125	1.618	0.274	2.060	2.125	200–310
R245fa	1.593	0.596	6.880	0.780	230–395
R236fa	2.410	0.554	5.089	0.678	270–370
R227ea	4.224	0.260	4.985	0.312	230–345
R1123	2.963	0.199	8.659	1.002	230–300
R1234yf	1.150	0.314	4.402	0.526	250–331
R1234ze(E)	3.016	0.555	3.518	2.286	250–351
R1225ye(Z)	1.018	0.260	3.343	4.508	250–355
R1336mzz(Z)	3.287	0.265	1.226	0.353	325–415
R1233zd(E)	2.936	0.506	9.172	1.342	250–400
R1224yd(Z)	1.358	0.237	1.114	1.585	280–375
Testing Set					
R32	0.565	0.225	5.821	2.827	200–316
R134a	1.443	0.389	7.415	2.440	200–344
R1243zf	1.092	0.295	5.439	0.874	270–345
average	1.899	0.405	4.753	1.539	

a The deviations are computed using parameters obtained with standard parametrization procedure and ANN-predicted.

b Experimental vapor pressure from refs (45, 74−79).

c Experimental saturated densities from refs (45, 74, 75, 77, 79−82).

**Figure 8:**
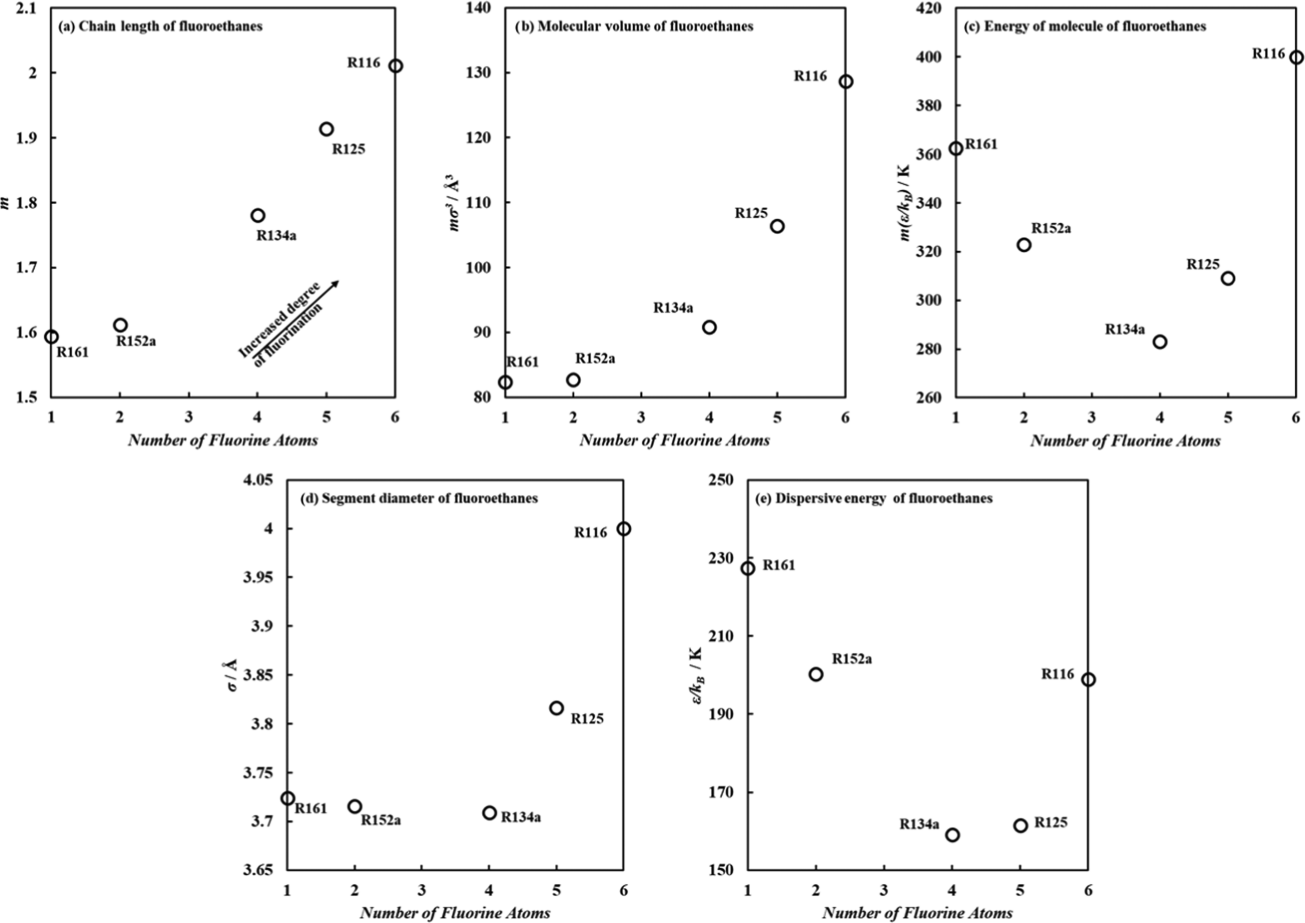
Trend of ANN-predicted molecular parameters for fluoroethanes as a function of the number of fluorine atoms from 1 to 6 atoms for R161–R116, for (a) chain length, (b) molecular volume, (c) energy of molecule, (d) segment diameter, and (e) dispersive energy.

To assess the adequacy of the training and testing of the ANN model, the trend of the predicted molecular parameters for fluoroethanes as a function of the number of fluorine atoms in the molecule was examined in Figure 8, starting from R161 with one fluorine atom up to hexafluoroethane (R161) with 6 fluorine atoms. Note that R161 was not previously fitted using the standard parametrization procedure, and its molecular parameters were fully predicted from the ANN model, using input parameters included in Table S1 in the SI. It can be seen that the increase in the degree of fluorination was accompanied by an increase in the chain length and molecular volume of fluoroethanes (see Figure 8a,b). This increase was rather minimal at a low degree of fluorination as seen with R161 and R152a, subsequently becoming more substantial with a higher number of fluorine atoms in the molecule. This can also be elucidated from the marginal change in segment diameter up to R134a in Figure 8d. On the other hand, the increased number of fluorine atoms had the opposite effect on the energy of the molecules, with reduced energy of the molecule and dispersive energy (see Figure 8c,e) with increasing number of fluorine atoms up to R134a. This is associated with the role of fluorine atoms in increasing the asymmetrical charge distribution in these molecules giving rise to higher dipolar contributions, seen earlier in Figure 5. The subsequent increase in dispersive energy for R125 and R116 is due to a higher contribution for dispersive interactions, due to a balanced electronegativity with a symmetrical number of fluorine atoms in the molecular structure, leading to a neutral R116 without the presence of a dipole moment. The trends obtained from ANN-predicted molecular parameters qualitatively agree with the observations highlighted in Figure 5.

**Figure 9:**
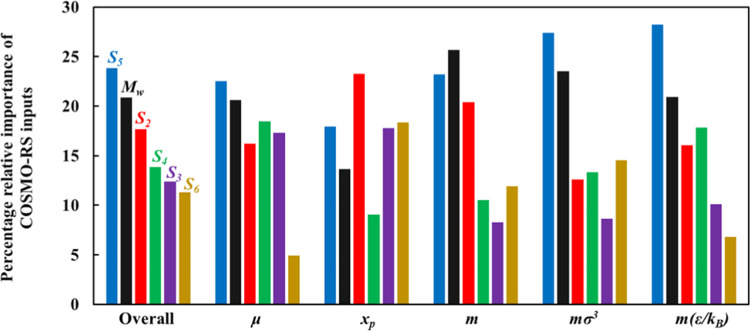
Relative importance (%) of ANN inputs for predicting each polar soft-SAFT output parameters and in overall terms.

For the developed ANN, the relative importance of each input parameter and usefulness in predicting the polar soft-SAFT output parameters are provided in Figure 9. It can be seen that for predicting the dipole moment, chain length, molecular volume, and molecular dispersive energy, the most important features are *S*_5_ (denoting slightly negative regions) and the molecular weight. The connection between the molecular weight and the chain length and molecular volume has been highlighted previously in Figure 5a. The same can also be established for the importance of *S*_5_ input feature, as it has the largest magnitude among all COSMO-RS inputs, related to the degree of fluorination, being the most influential factor on the intermolecular interactions of the refrigerants. In the case of the fraction of polar segments, the importance of *S*_2_ feature is the largest. Overall, both *S*_5_ and molecular weight have the largest impact on correlating the polar soft-SAFT parameters, while the remaining input features have a similar level of importance ≈15%.

### 3.4 Prediction of Thermophysical Properties of Refrigerants using the Developed ANN

An ultimate test for assessing the adequacy of the polar soft-SAFT molecular parameters predicted by the ANN was performed using these parameters for the needed thermodynamic characterization of selected refrigerants, including coexisting densities, vapor pressure, single-phase density, heat capacity, enthalpy of vaporization, and speed of sound. This is a rigorous testing framework applicable for any thermodynamic model, and at the same time, showcases the versatility of polar soft-SAFT as a holistic thermodynamic model.

The thermodynamic behavior using ANN-predicted parameters was compared to those obtained using standard parametrization (*i.e.*, fine-tune fitting to experimental data), and the available experimental data and results are presented in Table 4. The chosen refrigerants comprise R32, R134a, R1243zf, R236fa, and R1223zd(E), inclusive of different molecular features and refrigerants included in the training and testing sets.

**Table 4:** Polar Soft-SAFT Molecular Parameters for Selected Refrigerants Using Standard Parametrization and the ANN Model Developed in This Work

	standard parametrization^36,38^					ANN predicted				
compound	*m*	σ [Å]	ε/*k*_B_ [K]	μ·10^–30^ [C·m]	*x*_p_	*m*	σ [Å]	ε/*k*_B_ [K]	μ·10^–30^ [C·m]	*x*_p_
R32	1.376	3.506	164.5	6.598	0.75	1.427	3.541	167.9	6.635	0.75
R134a	1.813	3.770	169.5	6.865	0.70	1.780	3.709	159.1	6.908	0.70
R236fa	2.056	4.012	172.4	6.611	0.90	2.074	3.990	173.4	6.558	0.90
R1243zf	1.904	3.880	170.0	8.169	0.50	1.842	3.982	187.5	8.191	0.50
R1233zd(E)	2.331	3.819	232.6	3.812	0.80	2.393	3.759	220.9	3.740	0.80

**Figure 10:**
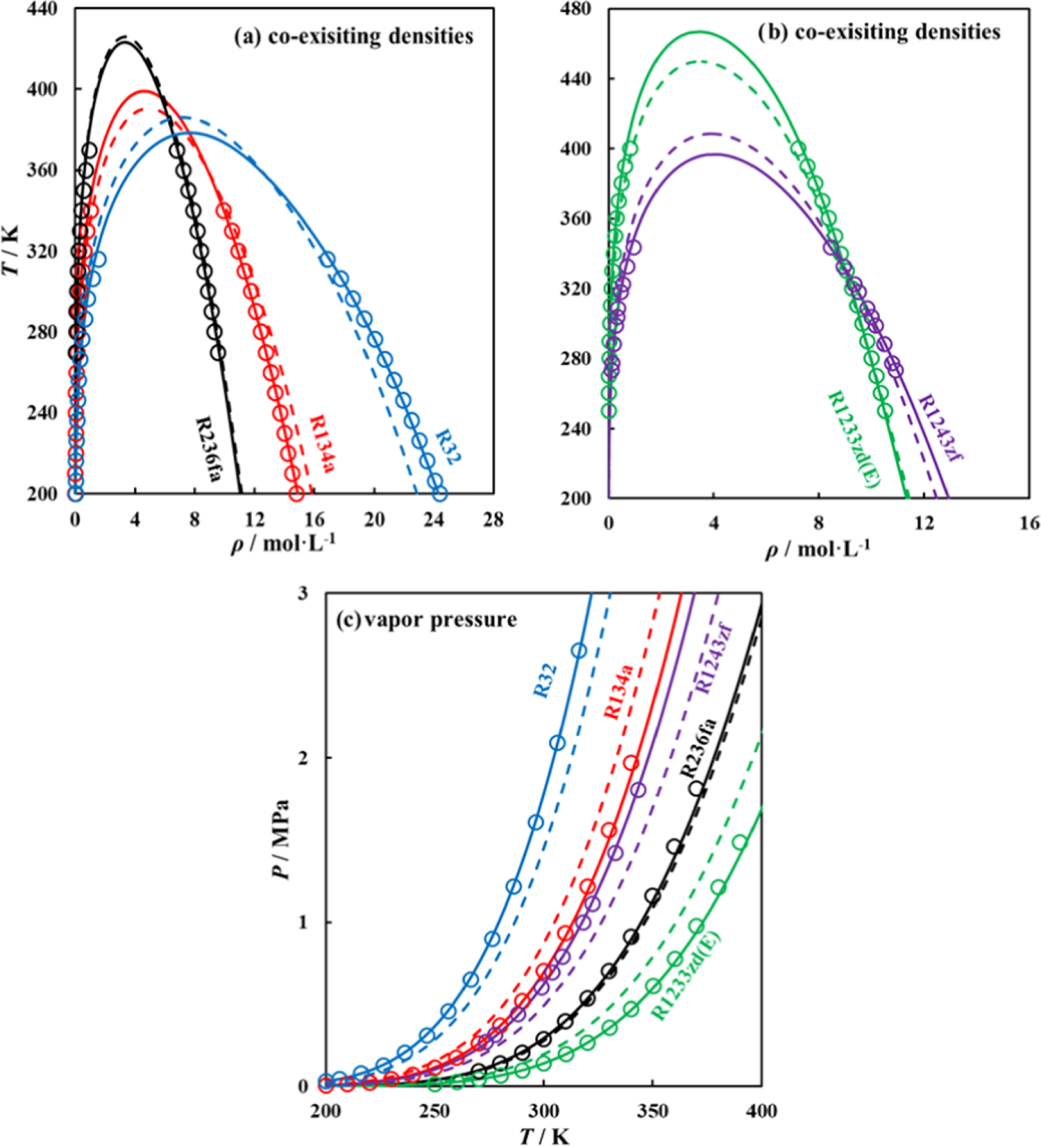
(a, b) Coexisting densities, and (c) vapor pressure for selected refrigerants obtained using polar soft-SAFT EoS, with fitted parameters (solid lines), ANN-predicted parameters (dashed lines), and experimental data (symbols). See text for details.

Shown in Figure 10, is the performance of fitted and predicted polar soft-SAFT parameters in computing coexisting densities^45,77,80^ and vapor pressures^45,76,77^ of the selected refrigerants, as compared to available experimental data. Better agreement of the fitted parameters, as opposed to the predicted ones, is expected as these experimental data are included in the fitting procedure to obtain them. Nonetheless, the ANN-predicted parameters capture the trend with a good agreement with experimental data with deviations for coexisting densities (AAD = 1.632%), and vapor pressure (AAD = 6.587%). These deviations are satisfactory considering that these parameters were predicted from the ANN without using any experimental data. The most consistent trends between fitted and predicted parameters were obtained for the R236fa refrigerant, as the training set was more biased to refrigerants with similar molecular structures.

**Figure 11:**
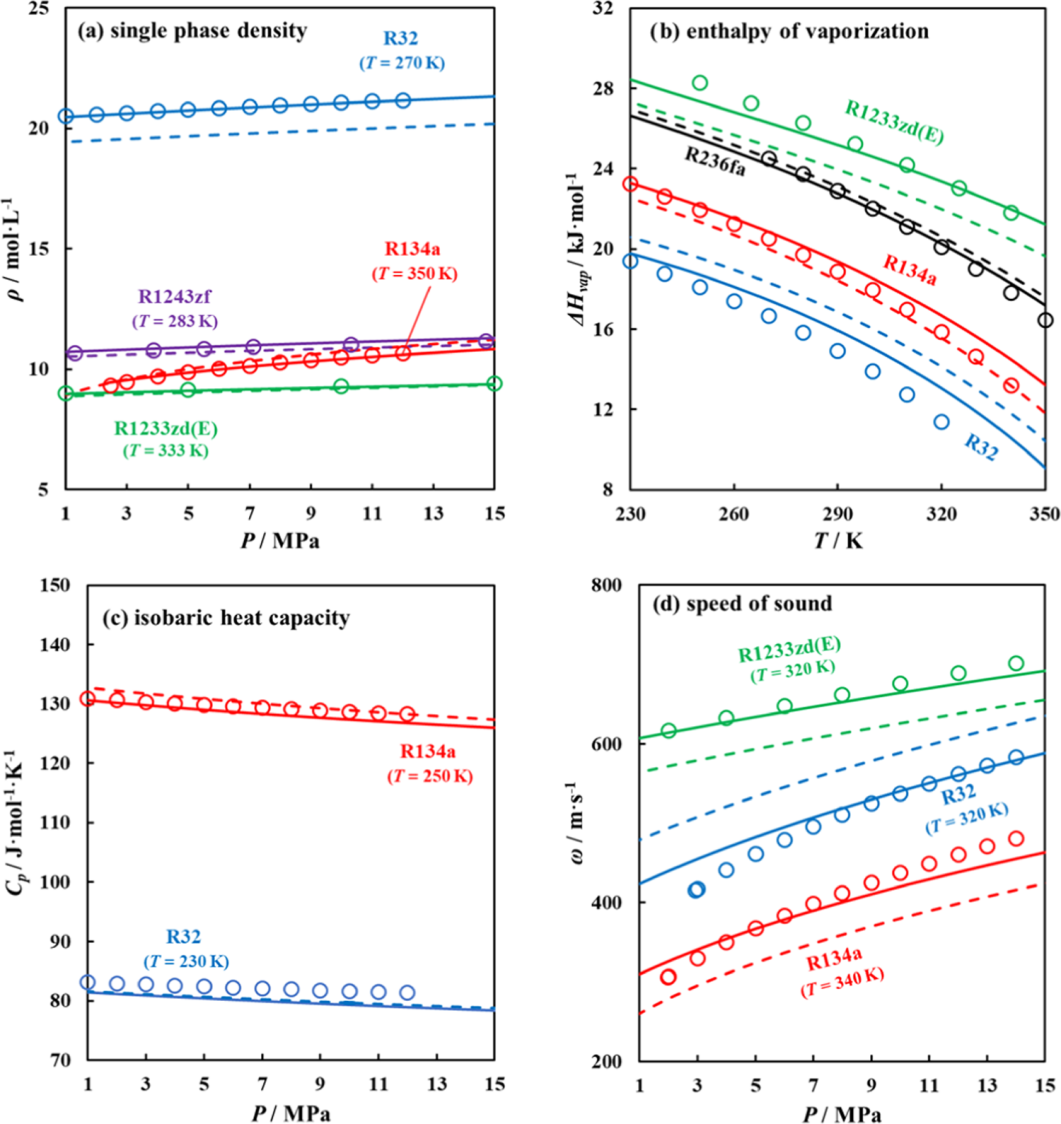
Other predicted thermodynamic properties include (a) single-phase density, (b) enthalpy of vaporization, (c) isobaric heat capacity, and (d) speed of sound for selected refrigerants predicted using polar soft-SAFT EoS, with fitted parameters (solid lines), ANN-predicted parameters (dashed lines), and experimental data (symbols).

The second stage of evaluating the adequacy of ANN-predicted polar soft-SAFT parameters is by examining other thermodynamic properties not included in the standard parametrization approach. Obtaining these properties is considered a stringent criterion in evaluating any thermodynamic model, as first and second-order derivative properties are very sensitive to small errors in modeling phase equilibria of pure fluids. As demonstrated in Figure 11, the trends using the fitted parameters calculated with the ANN-predicted parameters are consistent with the experimental data^45,83−85^ for properties such as single-phase density (AAD_ANN-MPs_ = 3.488%) and isobaric heat capacity (AAD_ANN-MPs_ = 1.311%).

A better agreement with experimental data^45,83−85^ was obtained using fitted parameters rather than ANN-predicted parameters in the cases of enthalpy of vaporization (AAD_ANN-MPs_ = 5.981%); owing to deviations in the predicted vapor pressures and speed of sound (AAD_ANN-MPs_ = 10.525%) as this property encompasses other derivative properties (*i.e.*, isobaric and isochoric heat capacities, and inverse reduced bulk modulus), increasing its sensitivity to errors arising from those properties.^86^

**Figure 12:**
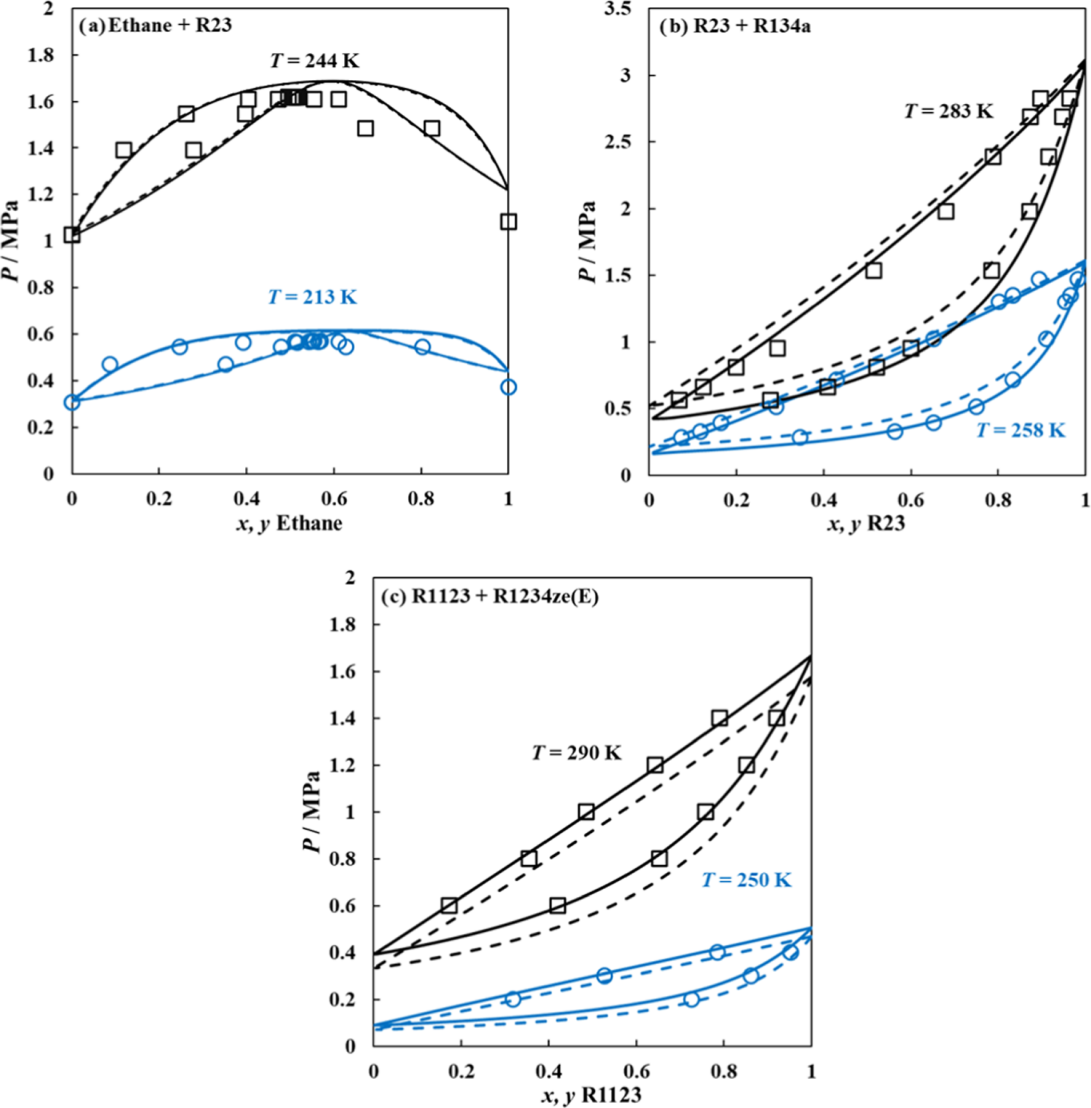
Binary mixtures for (a) ethane + R23, (b) R23 + R134a, and (c) R1123 + R1234ze(E) predicted using polar soft-SAFT EoS, with fitted parameters (solid lines), ANN-predicted parameters (dashed lines), and experimental data (symbols).

The last examination of the adequacy of ANN-predicted parameters is through their extension to computing vapor–liquid equilibria (VLE) of binary mixtures such as *n*-alkane + HFC, HFC + HFC, and HFO + HFO for selected mixtures to ensure the accurate quantification of dipolar interactions in refrigerants.^68,70^ The examined mixtures include R23 + ethane (modeled as a chainlike LJ fluid, using parameters included in Table S1 in the SI),^87^ R23 + R134a, and R1123 + R1234ze(E), with polar soft-SAFT employed in a predictive manner (binary interaction parameters kept at unity). Using the fitted parameters as opposed to the ANN-predicted parameters proved to be more accurate in capturing the reported experimental/molecular simulation behavior of these binary mixtures^88−90^ as shown in Figure 12. The errors with computing the VLE of these mixtures are due to the errors in the pure refrigerant vapor pressure, aside from that the trends are rather consistent as seen from the correct prediction of azeotropic compositions in the case of the ethane + R32 binary mixture. The error propagation decreases with the reducing temperature of the VLE, as the ANN-predicted parameters were more accurate at low-temperature regions rather than high-temperature regions.

The propagation of error in estimating additional thermodynamic properties and VLE of binary mixtures with ANN-predicted parameters stems from the errors seen earlier for coexisting densities and vapor pressure. This limitation can be attributed to the narrow training set used in ANN development, with expected enhanced accuracy with a larger training dataset. However, these errors are within an acceptable level of accuracy needed for the rapid screening of alternative eco-friendly refrigerants based on technical efficacy, guiding where the experimental efforts should be put for a detailed characterization of the most promising refrigerants.

## 4 Conclusions

In this work, a novel integrated approach was developed using a machine learning algorithm to map the relationships between molecular descriptors obtained from COSMO-RS with the molecular parameters required for obtaining the needed thermodynamic properties of refrigerants using the polar soft-SAFT equation of state.

With this approach, a direct link was established between specific molecular features of emerging sustainable refrigerants and their corresponding polar soft-SAFT molecular parameters. This enabled the acquisition of parameters for the equation of state for emerging systems even in the absence of experimental data, which effectively filled the gap in thermodynamic data required for evaluating their technical performance for different cooling applications.

The accuracy of the trained machine learning algorithm was verified with various statistical approaches. Additional tests were conducted on the adequacy of the ANN-predicted parameters in modeling thermodynamic properties from polar soft-SAFT with acceptable levels of errors versus the available experimental data in the range of 1.3–10.5% for properties such as coexisting densities, vapor pressure, enthalpy of vaporization, isobaric heat capacity, and speed of sound. Moreover, the ANN-predicted molecular parameters proved to be capable of characterizing the VLE behavior of selected binary mixtures with acceptable deviations. These deviations were attributable to the limited dataset used in the development of the machine learning model, which can be enhanced using large datasets, requiring a library of polar soft-SAFT molecular models for a larger variety of systems.

Despite the already mentioned limitations, the results in this work showcase the potentiality of this novel integrated approach for the first technical evaluation of newly developed refrigerants, even in the absence of sufficient experimental data, facilitating the search for green alternatives meeting technical and environmental requirements.

## Supporting Information Available

ANN input σ-profiles discretized COSMO-RS descriptors (Table S1); the output polar soft-SAFT molecular parameters (Tables S2 and S3); and ANN weights and biases (Tables S4–S6) (PDF)
