# Supplementary material for: Searching for Sustainable Refrigerants by Bridging Molecular Modeling with Machine Learning
Source: Ind Eng Chem Res. 2022 May 18;61(21):7414–29. doi: 10.1021/acs.iecr.2c00719 (PMC9165071; doi:10.1021/acs.iecr.2c00719)
Supplement: Supplementary file 1 — ie2c00719_si_001.pdf [file ie2c00719_si_001.pdf]

Supporting Information

Searching for Sustainable Refrigerants by Bridging Molecular Modeling with Machine Learning

Ismail I.I. Alkhatib[a,b], Carlos G. Albà[c], Ahmad S. Darwish[b], Fèlix Llovell[c], Lourdes F. Vega[a,b*]

[a] Research and Innovation Center on CO_2 and Hydrogen (RICH), Khalifa University, PO Box 127788, Abu Dhabi, United Arab Emirates.
[b]Chemical Engineering Department, Khalifa University, PO Box 127788, Abu Dhabi, United Arab Emirates.
[c] Department of Chemical Engineering, ETSEQ, Universitat Rovira i Virgili (URV), Av. Països Catalans 26, 43007, Tarragona, Spain.
[*] Corresponding authors e-mail addresses: lourdes.vega@ku.ac.ae

Table S1. Input dataset for ANN with molar mass and COSMO-RS molecular refrigerants included in this work.

Compound	*Mw (g.mol^{-1})*	S_2	S_3	S_4	S_5	S_6
**R41**	34.03	0.0000	0.0118	0.0205	0.0053	0.0158
**R32**	52.02	0.0000	0.0208	0.0061	0.0246	0.0064
**R23**	70.01	0.0070	0.0062	0.0065	0.0536	0.0000
**R161**	48.06	0.0000	0.0079	0.0336	0.0112	0.0169
**R152a**	66.05	0.0000	0.0210	0.0186	0.0219	0.0107
**R134a**	102.03	0.0022	0.0146	0.0108	0.0599	0.0023
**R125**	120.02	0.0082	0.0046	0.0155	0.0695	0.0000
**R245fa**	134.05	0.0039	0.0170	0.0137	0.0741	0.0015
**R236fa**	152.04	0.0082	0.0074	0.0165	0.0879	0.0000
**R227ea**	170.03	0.0080	0.0029	0.0254	0.0792	0.0000
**R1123**	82.02	0.0018	0.0065	0.0233	0.0496	0.0002
**R1243zf**	96.05	0.0001	0.0186	0.0178	0.0627	0.0000
**R1234yf**	114.04	0.0003	0.0124	0.0268	0.0636	0.0000
**R1234ze(E)**	114.04	0.0038	0.0103	0.0216	0.0684	0.0000
**R1225ye(Z)**	132.03	0.0040	0.0055	0.0290	0.0705	0.0000
**R1336mzz(Z)**	164.06	0.0062	0.0067	0.0324	0.0860	0.0000
**R1233zd(E)**	130.49	0.0038	0.0087	0.0296	0.0725	0.0000
**R1224yd(Z)**	149.49	0.0035	0.0046	0.0375	0.0725	0.0000
**R116**	138.01	0.0005	0.0117	0.0210	0.0093	0.0000

Table S2. Polar soft-SAFT molecular parameters for refrigerants included in this work.

Class	Compound	m	σ [Å]	ε/k_B [K]	$\mu\cdot10^{-30}$ [C·m]§	x_p	Ref.
**Alkane**	Ethane	1.392	3.756	202.5	-	-	[1]
**3rd gen. HFCs**	R41	1.371	3.400	180.3	6.17427	0.50	[2]
	R32	1.376	3.506	164.5	6.59790	0.75	[2]
	R23	1.397	3.610	147.9	5.50047	0.90	[2]
	R161	1.577	3.693	232.3	6.47014	0.33	[2]
	R152a	1.662	3.754	202.3	7.54522	0.50	[2]
	R134a	1.813	3.770	169.5	6.86475	0.70	[3]
	R125	1.887	3.790	165.1	5.21360	0.90	[2]
	R245fa	2.479	3.675	197.1	5.16690	0.80	[2]
	R236fa	2.056	4.012	172.4	6.61124	0.90	[2]
	R227ea	2.131	4.033	190.7	4.85669	1.00	[2]
**4th gen. HFOs**	R1123	1.527	3.760	175.3	5.73730	0.80	[2]
	R1243zf	1.904	3.880	170.0	8.16890	0.50	[2]
	R1234yf	1.740	4.082	191.6	6.70790	0.70	[3]
	R1234ze(E)	2.044	3.821	204.0	4.80330	0.75	[2]
	R1225ye(Z)	2.077	3.845	172.4	6.03750	0.80	[2]
	R1336mzz(Z)	1.806	4.430	195.6	10.6406	0.60	[2]
**4th gen. HCFOs**	R1233zd(E)	2.331	3.819	232.6	3.81190	0.80	[2]
	R1224yd(Z)	2.278	3.899	202.4	5.63720	0.85	[2]

§Experimental dipole moment[4–9]

Table S3. Output dataset for ANN with polar soft-SAFT molecular parameters for refrigerants included in this work.

Compound	$\mu\cdot10^{-30}$ [C·m]§	x_p	m	$m\sigma^3$ [Å^3]	$m\varepsilon/k_B$ [K]
R41	6.174	0.500	1.371	53.886	247.191
R32	6.598	0.750	1.376	59.300	226.352
R23	5.500	0.900	1.397	65.723	206.616
R161	6.470	0.330	1.577	79.427	366.337
R152a	7.545	0.500	1.662	87.925	336.223
R134a	6.865	0.700	1.813	97.145	307.304
R125	5.214	0.900	1.887	102.728	311.544
R245fa	5.167	0.800	2.479	123.041	488.611
R236fa	6.611	0.900	2.056	132.772	354.454
R227ea	4.857	1.000	2.131	139.787	406.382
R1123	5.737	0.800	1.527	81.171	267.683
R1243zf	8.169	0.500	1.904	111.215	323.680
R1234yf	6.708	0.700	1.740	118.350	333.384
R1234ze(E)	4.803	0.750	2.044	114.028	416.976
R1225ye(Z)	6.038	0.800	2.077	118.066	358.075
R1336mzz(Z)	10.641	0.600	1.806	157.011	353.254
R1233zd(E)	3.812	0.800	2.331	129.835	542.191
R1224yd(Z)	5.637	0.850	2.278	135.025	461.067

Table S4. ANN weights and bias for the links between neurons in the input layer, and neurons in the 1st hidden layer.

		Neurons in input layer (i)						***Neuron bias***
		Mw	S_2	S_3	S_4	S_5	S_6	(b_{ij})
**Neurons in 1st hidden layer (j)**	μ_1	0.0199	759.393	-221.629	-53.3194	-28.989	-55.8245	-0.945
	μ_2	0.0599	273.925	29.648	31.886	44.855	-1.577	-10.543
	μ_3	-0.00879	219.773	-202.017	-46.3872	19.301	-10.375	0.764
	μ_4	0.0668	-691.009	-127.769	103.573	3.469	-59.979	-5.633
	μ_5	0.000495	127.437	-3.887	-45.675	-30.769	337.681	1.913
	μ_6	0.00394	800.905	-15.216	93.798	57.655	-72.769	-8.912
	μ_7	-0.0315	521.543	330.952	22.345	1.337	146.780	-3.278
	μ_8	0.0449	-207.964	-48.581	80.760	2.153	-99.433	-2.484
	μ_9	0.0220	538.290	-139.335	-95.831	-40.209	-4.413	-0.301
	x_{p1}	0.0437	59.978	-134.377	-172.845	3.124	49.577	-0.0166
	x_{p2}	-0.0382	119.168	88.579	139.689	41.318	7.879	-1.839
	x_{p3}	-0.0139	-388.942	91.501	-16.887	-26.252	33.729	2.668

Table S5. ANN weights and bias for the links between neurons in the 1st hidden layer, and neurons in the 2nd hidden layer.

		Neurons in 2nd hidden layer (k)			
		H_1	H_2	H_3	H_4
**Neurons in 1st hidden layer (j)**	μ_1	-0.0196	0.0292	0.0132	-0.0759
	μ_2	-0.0186	-0.0014	-0.0313	0.0094
	μ_3	0.0869	0.0084	-0.0862	0.0653
	μ_4	-0.0445	0.0294	-0.0162	-0.0631
	μ_5	0.0471	-0.0016	0.0625	-0.0085
	μ_6	-0.0603	-0.0115	0.0047	-0.0019
	μ_7	-0.0109	-0.0379	0.0722	0.0284
	μ_8	-0.1219	-0.0184	-0.0273	-0.0020
	μ_9	-0.0359	-0.0017	0.0258	-0.0207
	x_{p1}	0.0020	0.0144	-0.0121	-0.0206
	x_{p2}	-0.0001	0.0076	-0.0503	-0.1121
	x_{p3}	0.0300	0.0151	0.0457	-0.0402
Neuron bias (b_{jk})		0.0973	-0.0019	0.0408	0.0209

Table S6. ANN weights and bias for the links between neurons in the output layer, and neurons in the 1st or 2nd hidden layers.

		Neurons in output layer (O)				
		$\mu \cdot 10^{-30}$	x_p	m	$m\sigma^3$	$m\varepsilon/k_B$
**Neurons in 1st hidden layer (j)**	μ_1	11.898	-	-	-	-
	μ_2	-0.114	-	-	-	-
	μ_3	-8.478	-	-	-	-
	μ_4	-1.612	-	-	-	-
	μ_5	-1.039	-	-	-	-
	μ_6	-3.692	-	-	-	-
	μ_7	-0.111	-	-	-	-
	μ_8	1.618	-	-	-	-
	μ_9	4.457	-	-	-	-
	x_{p1}	-	-0.524	-	-	-
	x_{p2}	-	-0.804	-	-	-
	x_{p3}	-	-0.482	-	-	-
Neurons in 2nd hidden layer (k)	H_1	-	-	-100.481	1358.022	-42068.548
	H_1	-	-	411.074	-8831.445	178751.003
	H_1	-	-	119.880	-2067.174	51621.750
	H_1	-	-	226.605	-4999.385	96884.334
Neuron bias (b_{jo})		2.699	0.889	2.073	102.779	430.899

References

(1) Pàmies, J. C.; Vega, L. F. Vapor–Liquid Equilibria and Critical Behavior of Heavy *n* -Alkanes Using Transferable Parameters from the Soft-SAFT Equation of State. *Ind. Eng. Chem. Res.* **2001**, *40* (11), 2532–2543. https://doi.org/10.1021/ie000944x.

(2) Albà, C. G.; Alkhatib, I. I. I.; Llovell, F.; Vega, L. F. Assessment of Low Global Warming Potential Refrigerants for Drop-In Replacement by Connecting Their Molecular Features to Their Performance. *ACS Sustain. Chem. Eng.* **2021**, *9* (50), 17034–17048. https://doi.org/10.1021/acssuschemeng.1c05985.

(3) Albà, C. G.; Llovell, F.; Vega, L. F. Searching for Suitable Lubricants for Low Global Warming Potential Refrigerant R513A Using Molecular-Based Models: Solubility and Performance in Refrigeration Cycles. *Int. J. Refrig.* **2021**, *128*, 252–263. https://doi.org/10.1016/j.ijrefrig.2021.04.010.

(4) Lemmon, E. W.; Huber, M. L.; McLinden, M. O. *NIST Reference Fluid Thermodynamic and Transport Properties — REFPROP, Version 9.0, National Institute of Standards and Technology, Gaithersburg, MD*; 2013.

(5) Di Nicola, G.; Coccia, G.; Pierantozzi, M.; Tomassetti, S. Equations for the Surface Tension of Low GWP Halogenated Alkene Refrigerants and Their Blends. *Int. J. Refrig.* **2018**, *86*, 410–421. https://doi.org/10.1016/j.ijrefrig.2017.11.023.

(6) Fouad, W. A.; Vega, L. F. Next Generation of Low Global Warming Potential Refrigerants: Thermodynamic Properties Molecular Modeling. *AIChE J.* **2018**, *64* (1), 250–262. https://doi.org/10.1002/aic.15859.

(7) Raabe, G. Molecular Simulation Studies on the Vapor–Liquid Equilibria of the Cis - and Trans - HCFO-1233zd and the Cis - and Trans -HFO-1336mzz. *J. Chem. Eng. Data* **2015**, *60* (8), 2412–2419. https://doi.org/10.1021/acs.jced.5b00286.

(8) Hanwell, M. D.; Curtis, D. E.; Lonie, D. C.; Vandermeersch, T.; Zurek, E.; Hutchison, G. R. Avogadro: An Advanced Semantic Chemical Editor, Visualization, and Analysis Platform. *J. Cheminform.* **2012**, *4* (1), 17. https://doi.org/10.1186/1758-2946-4-17.

(9) Raabe, G. Molecular Simulation Studies on Refrigerants Past – Present – Future. *Fluid Phase Equilib.* **2019**, *485*, 190–198. https://doi.org/10.1016/j.fluid.2018.12.022.
